# New Approaches to Vaccine Adjuvants: Inhibiting the Inhibitor

**DOI:** 10.1371/journal.pmed.0030057

**Published:** 2006-01-03

**Authors:** Barney S Graham

## Abstract

Graham discusses a study that explores new approaches to enhancing vaccine-induced immune responses.

Development of an effective HIV-1 vaccine has been an elusive goal for over 20 years despite being an urgent global priority. The agonizingly slow progress is not from lack of effort, but is a consequence of the insidious biology of the virus. HIV-1 has many features that make vaccine development challenging, if not impossible [1].

## Obstacles to Vaccine Development

Pessimism is based in part on the empirical observation that there has never been a confirmed case of viral clearance and recovery from HIV-1 infection, and from the mounting evidence that HIV-1 superinfection (see Glossary) is common (in other words, if natural infection does not protect against infection with other HIV strains, why would we expect vaccination to offer protection?) [2].

**Glossary**

**gp120:** The extracellular portion of the HIV-1 Env glycoprotein responsible for binding to CD4^+^ and co-receptors.

**gp120-pulsed bone marrow–derived dendritic cells:** Dendritic cells are key antigen-presenting cells that in this case were derived from mouse bone marrow and expanded with interleukin-4 and granulocyte/monocyte colony stimulating factor in vitro before treatment with gp120.

**gp160:** Full length HIV-1 Env glycoprotein of 160 kD molecular weight responsible for attachment to and entry into target cells.

**IRF-3:** Transcription factor, interferon regulatory factor-3.

**JAKs:** JAKs associate with cytokine receptors, and are important for tyrosine phosphorylation of the receptor, of each other, and of signal transduction and activator-of-transcription molecules that participate in the signaling cascade from the cytokine receptors to the nucleus.

**LPS:** Lipopolysaccharide is analogous to endotoxin derived from Gram-negative bacteria.

**MyD88:** Myeloid differentiation factor 88.

**NF-κB:** Transcription factor, nuclear factor-κB.

**PAMP:** Pathogen-associated molecular pattern.

**PolyI:C:** Polyinosinic:polycytidylic acid is a synthetic molecule that mimics double-stranded RNA.

**R837:** (1-(2-methyl propyl)-1H-imidazo[4,5-c]quinolin 4-amine} is a TLR-7 and TLR-8 ligand.

**siRNA inhibition:** Small interfering RNAs are approximately 22 nucleotide-long RNA molecules that efficiently inhibit translation of their complementary mRNA.

**STAT:** Signal transduction and activator-of-transcription molecules associate in dimers after phosphorylation by JAKs, translocate to the nucleus, and promote transcription of selected genes.

**Superinfection:** Infection with a strain of HIV-1 that is genetically distinct from the HIV-1 present in a person with a stable immune response to the original infection.

**Th1 response:** A T helper 1 response implies a polarized CD4^+^ T cell response with dominant expression of interferon-&gamma;. In mice, this is associated with a predominant IgG2a antibody isotype response.

**Th2 response:** A T helper 2 response implies a polarized CD4^+^ T cell response with dominant expression of interleukin-4, interleukin-5, interleukin-9, and interleukin-13. In mice, this is associated with a predominant IgG1 antibody isotype response.

**TIR:** A cytoplasmic signaling domain on TLRs, Toll/interleukin-1 receptor–resistance domain.

**TLR-3:** Toll-like receptor 3 recognizes double-stranded RNA including the synthetic molecule polyI:C.

**TLR-4:** Toll-like receptor 4 recognizes LPS.

**TLR-7/8:** Toll-like receptors 7 and 8 recognize single-stranded RNA and some other ligands such as imiquimod and resiquimod.

**Toll-like receptor:** Family of proteins homologous to the Drosophila Toll receptor, found to have the capacity to recognize molecular patterns associated with pathogens.

**TRIF:** TIR domain-containing adapter-inducing interferon, also called TICAM-1 (Toll-IL-1 receptor homology domain-containing adapter molecule).

The high replication error rate of HIV-1 is the basis for two of the greatest challenges in vaccine design. First, extreme genetic diversity makes vaccine antigen selection difficult. Second, the high mutation rate provides many opportunities for the virus to escape vaccine-induced immune responses.

In addition, HIV-1 glycoprotein (gp)160 can directly cause dysfunction of antigen-presenting cells, and HIV-1 can infect CD4^+^ T cells, which cripples the key elements required to initiate the adaptive immune response to viral pathogens. After cells are infected, there are virus-specific mechanisms that disrupt the normal regulation of immune activation, and HIV-1 can also become latent or infect immunoprivileged sites and remain hidden from the immune response.

The list of barriers to vaccine development continues with the multitude of structural features of gp160 that evade antibody neutralization [3]. Therefore, an effective HIV-1 vaccine will need to induce immune responses that can swiftly respond to infection and efficiently clear or control infection at very low levels of replication. To do this may require better vaccine adjuvants or delivery vehicles than are currently available.

## A Novel Approach to Vaccine Development

In a new study in *PLoS Medicine*, Song et al. explore new approaches to enhancing vaccine-induced immune responses [4]. The field of vaccine adjuvants has been rapidly evolving since the discovery of the Toll-like receptor (TLR) family of pattern recognition molecules [5]. Traditionally, adjuvants have been developed empirically from natural substances found to cause inflammation. Since cytokines were found to be the effector molecules for many adjuvant effects [6], there has been an effort to build the optimal vaccine adjuvant effect one cytokine at a time.

However, this approach underestimated the complexity and timing of events necessary to augment immune responses, and has given way to using specific TLR ligands as vaccine adjuvants [7]. Activating immune responses at this level is analogous to using the original empirically derived adjuvants, but the molecular mechanisms are better defined. In the current study, Song and colleagues have achieved an even broader effect by inhibiting a natural inhibitor of TLR and cytokine signaling. The family of suppressor of cytokine signaling (SOCS) molecules targets Janus kinases (JAKs) and nuclear factor-κB (NF-κ B) pathways involved in transmitting signals from cytokine receptors and TLRs to the nucleus to control genes encoding mediators of inflammation (Figure 1) [8].

**Figure 1:**
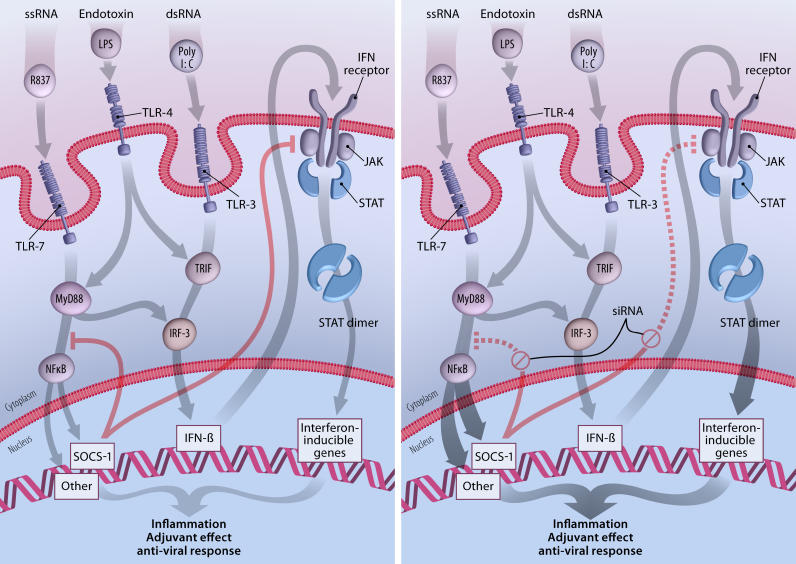
SOCS “Silencing” Enhances the Adjuvant Effect of TLR Ligands Substances common in viral pathogens (substances such as single-stranded RNA [ssRNA] and double-stranded RNA [dsRNA]) or in bacteria (substances such as endotoxin) are recognized by TLRs as pathogen-associated molecular patterns. When the TLRs are triggered, a series of signaling events occur that is simplified and schematized in this figure. These events lead to inflammation and activation of innate and adaptive immune responses. SOCS family members are also activated, and serve as an internal control to diminish the intensity and duration of inflammation. In the left-hand panel, R837 mimics ssRNA as a ligand for TLR-7, and initiates signaling through the MyD88 pathway, eventually resulting in the release of NF-κB and in the upregulation of SOCS1 and many genes involved in inflammation. PolyI:C is a synthetic mimic of dsRNA and triggers TLR-3-associated JAK/signal transduction and activator-of-transcription (STAT) signaling through TIR domain-containing adapter-inducing interferon (TRIF), activation of interferon regulatory factor (IRF)-3, and increased production of type 1 interferon. LPS can activate both pathways. SOCS1 specifically interferes with JAK2 and may also inhibit a step between MyD88 and NF-κB release into the nucleus, although this is controversial. This balanced internal feedback mechanism results in a controlled inflammatory process with adjuvant and antiviral effects. When SOCS1 production is blocked by siRNA (right-hand panel), the control of the inflammatory process is temporarily lost, leading to a greater adjuvant and antiviral response that appears to improve vaccine-induced immune responses. (Illustration: Giovanni Maki)

The authors found that small interfering RNA (siRNA) inhibition of SOCS1 in HIV-1 gp120-pulsed bone marrow–derived dendritic cells (DCs) improved the immune response to those DCs delivered as a carrier of vaccine antigen. In addition, SOCS1 inhibition improved the adjuvant effect of polyinosinic:polycytidylic acid (polyI:C), R837, or lipopolysaccharide (LPS) on the DC vaccine. These adjuvants are recognized by TLR-3, TLR-7/8, or TLR-4, respectively. SOCS1 inhibition increased the magnitude of the antibody response and the magnitude and lytic activity of the CD8^+^ T cell response. It also appeared to increase the duration of antibody and T cell responses. SOCS1 inhibition increased cytokine production from in vitro–stimulated DCs and also led to increased cytokine production from CD4^+^ T cells.

The pattern of increased cytokine production was broad and included proinflammatory cytokines, as well as cytokines traditionally associated with polarized T helper cell (Th)1 or Th2 responses. Importantly, in vivo delivery of plasmid DNA expressing the SOCS1 siRNA, together with a plasmid DNA vaccine encoding a modified HIV-1 envelope (Env) protein, improved Env-specific immune responses in mice, particularly when the polyI:C or the R837 adjuvant was administered following immunization.

## Implications of the Approach

We maintain a tenuous balance between adequate control of pathogens or neoplasms and excessive chronic inflammation (e.g., autoimmune diseases) and uncontrolled proliferation (e.g., lymphoma) of our immune system. There are more pathways and receptors devoted to controlling the immune response than to inducing immune responses. It is critical for health maintenance to have a highly evolved process for turning off immune responses when there are so many inflammatory challenges. Therefore, it is not surprising that enhancing immune responses by removing inhibition may be more potent than the typical adjuvant concept of actively stimulating immune responses. This has been noted before when evaluating the adjuvant effects of cytokines [9]. Song and colleagues' study has shown the potential to broadly augment immune activation triggered by vaccine antigens and a variety of adjuvants. Their approach allows the antigen-presenting cell to determine the composition and kinetics of effector molecules and the co-stimulation needed for the development of potent adaptive immune responses.

Before considering this approach for clinical use, it will be important to determine whether the immune response enhancement seen in mice can be achieved in higher-order animals such as nonhuman primates. The safety of this approach will need to be more fully defined in species other than mice. It will be important to show that SOCS inhibition is temporary, and that normal regulation of immune responses is maintained following the vaccination. This may require long-term evaluation in multiple species to assure there is not excessive or prolonged inflammation. Identifying pharmacological inhibitors of SOCS family members may provide a more temporally controlled approach for releasing the inhibition of TLR and cytokine signaling, and provide an additional safeguard against the theoretical risk of chronic inflammation. As the authors suggest, waking up the immune system by inhibiting the inhibitors may have therapeutic benefits in some settings.

The struggle to develop a preventive vaccine for HIV-1 has taught us much of what we know about immunology, and will continue to have benefits at the conceptual level and for antiviral vaccine development in general. HIV-1 vaccine development will be facilitated by innovative approaches to vaccine formulation and delivery such as SOCS “silencing” that improve the kinetics, the magnitude, and the composition of immune responses. However, ultimate success will depend on the design of vaccine antigens that can elicit the right antibody and T cell specificity to achieve virus neutralization and clearance.

## Abbreviations

DC: dendritic cell
Env: envelope
gp: glycoprotein
JAK: Janus kinase
LPS: lipopolysaccharide
NF-κ,B: nuclear factor-κB
polyI: C: polyinosinic:polycytidylic acid
siRNA: small interfering RNA
SOCS: suppressor of cytokine signaling
Th: T helper cell
TLR: Toll-like receptor
